# Supplementary material for: Chronically Radiation-Exposed Survivor Glioblastoma Cells Display Poor Response to Chk1 Inhibition under Hypoxia
Source: Int J Mol Sci. 2022 Jun 24;23(13):7051. doi: 10.3390/ijms23137051 (PMC9266388; doi:10.3390/ijms23137051)
Supplement: Supplementary file 1 [file ijms-23-07051-s001.zip › ijms-1709020-supplementary.pdf]

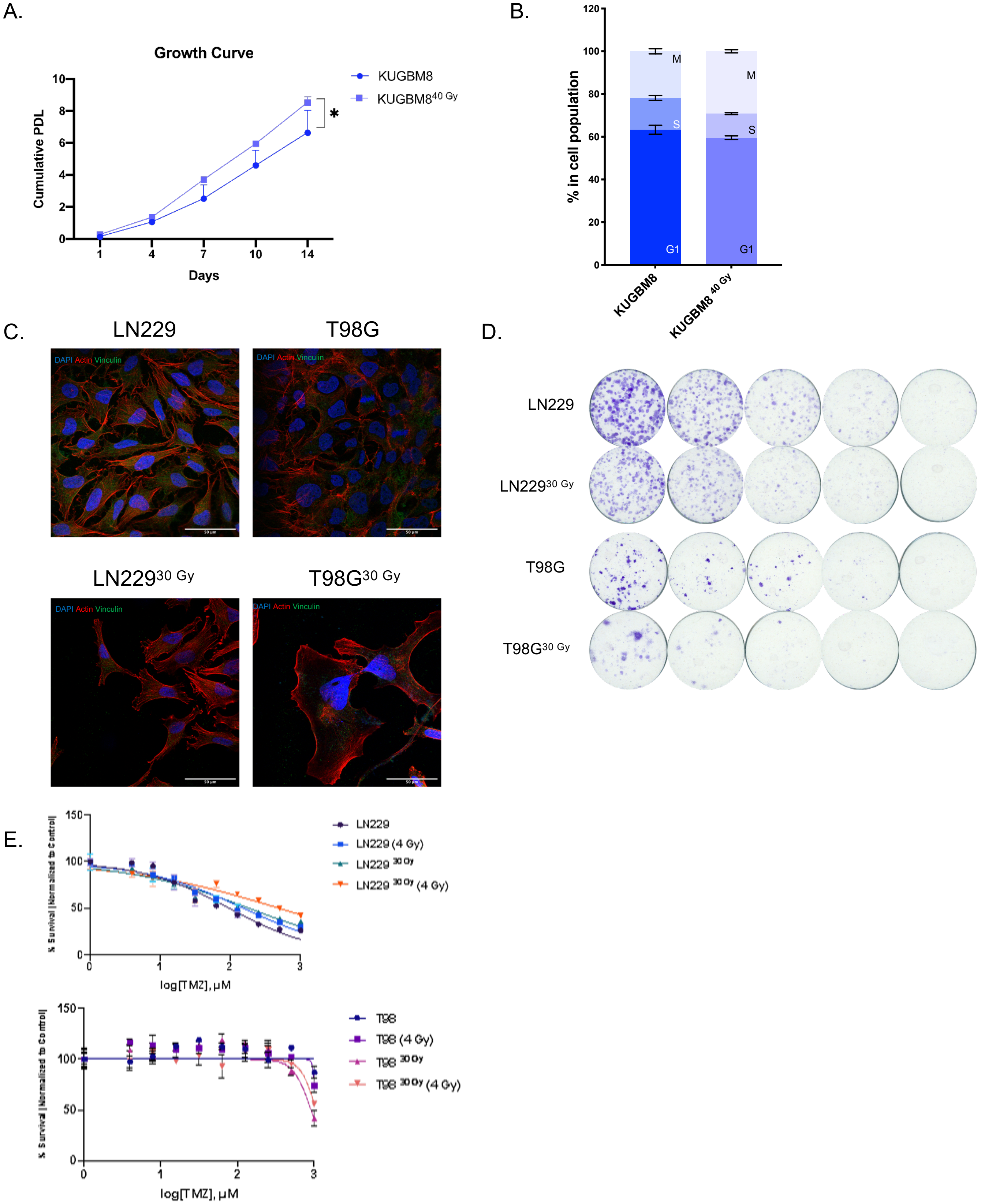

Supplementary Figure S1. A. Proliferation rates of KUGBM8 and KUGBM8$^{40\ Gy}$ cells. **B.** Cell cycle distributions of KUGBM8 and KUGBM8$^{40\ Gy}$ cells. **C.** Immunofluorescence staining for T98G and LN229 cells and their IR-Surv populations (DAPI: blue, Actin: red, Vinculin: Green). **D.** Representative images of clonogenic assay upon increasing doses of IR of T98G and LN229 cell lines. **E.** Dose-response curves of naïve and IR-Surv T98G and LN229 cells upon TMZ, IR or combination treatment.

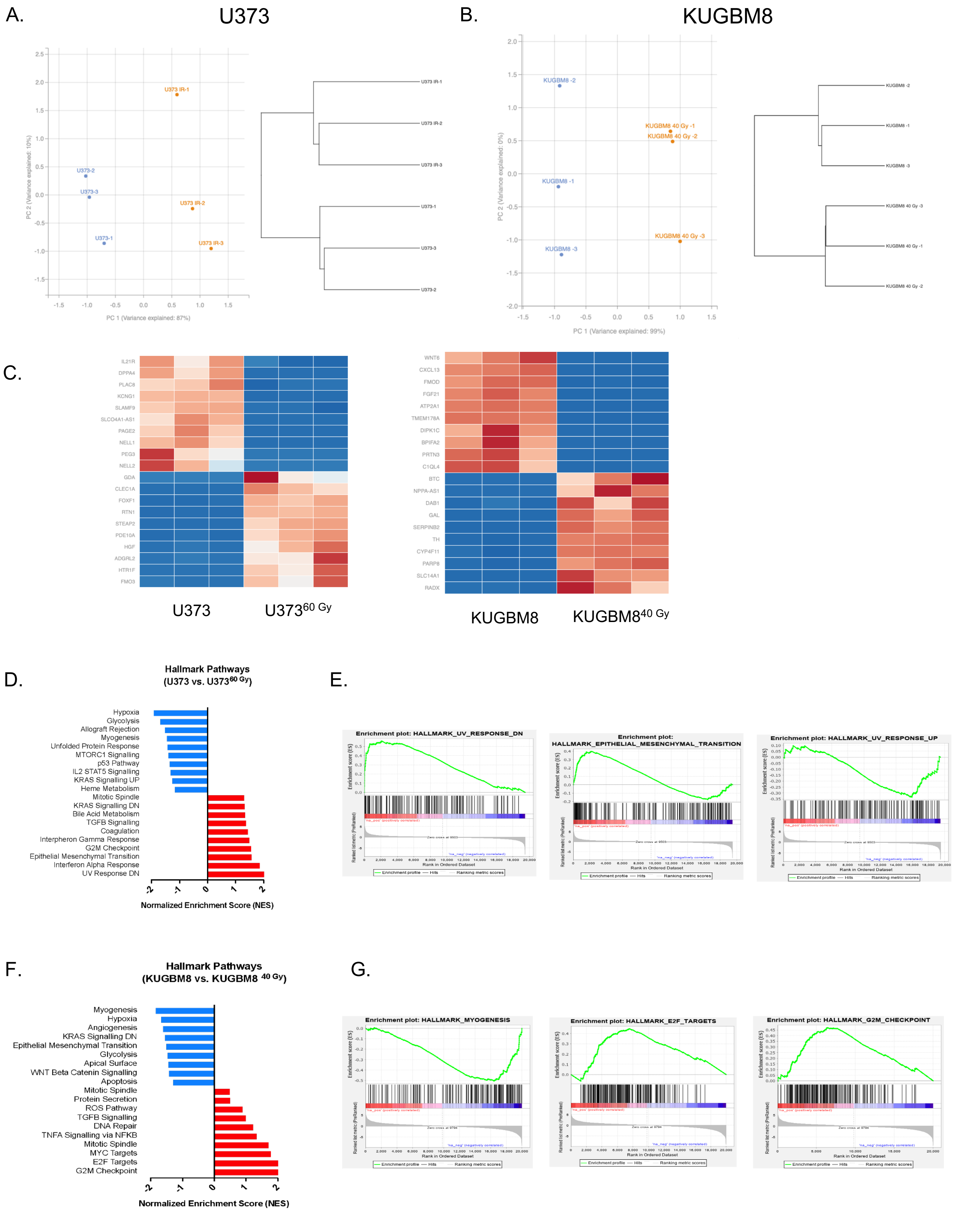

Supplementary Figure S2. A-B. Principal component analysis (PCA) plots and representations of hierarchical clustering of U373-U373$^{60\ Gy}$ and KUGBM8-KUGBM8$^{40\ Gy}$ samples. **C.** Heatmap of top 10 differentially upregulated and downregulated genes in U373-U373$^{60\ Gy}$ and KUGBM8-KUGBM8$^{40\ Gy}$ cells. **D-E.** Hallmark GSEA and representative enrichment plots of U373 cells. **F-G.** Hallmark GSEA and representative enrichment plots of KUGBM8 cells.

A.

Hallmark Hypoxia

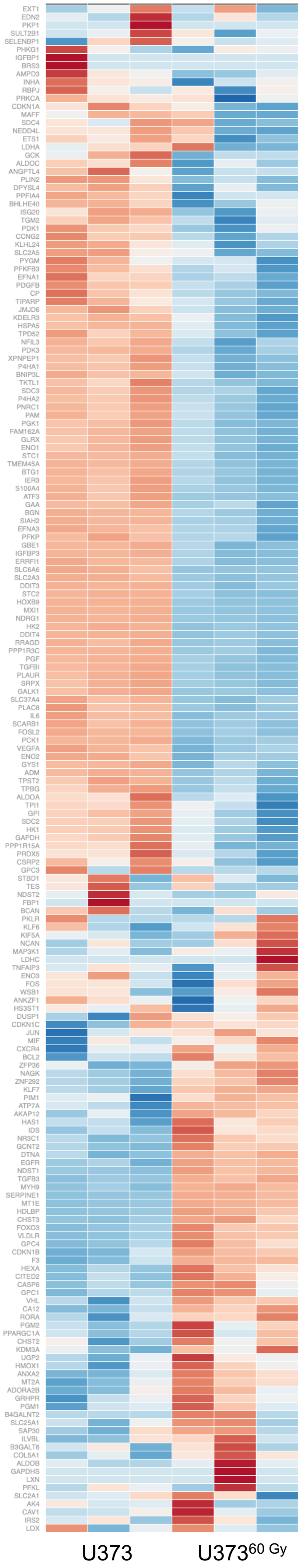

Gene	Log$_2$(FC)	Gene	Log$_2$(FC)
KDM3A	-0,02	ISG20	-0,97
PGM1	-0,02	ATF3	-0,98
SLC25A1	-0,03	SRPX	-1,00
MAP3K1	-0,03	SLC6A6	-1,01
GRHPR	-0,04	NFIL3	-1,01
KLF6	-0,06	FAM162A	-1,05
ILVBL	-0,07	TPST2	-1,05
COL5A1	-0,07	TGM2	-1,05
SAP30	-0,08	GBE1	-1,07
CHST2	-0,09	ALDOC	-1,07
PIM1	-0,10	ENO2	-1,10
B3GALT6	-0,11	GALK1	-1,11
CAV1	-0,12	EFNA3	-1,11
WSB1	-0,12	STC2	-1,16
DUSP1	-0,12	SLC2A5	-1,17
JUN	-0,12	PDK3	-1,20
IRS2	-0,14	ADM	-1,20
AK4	-0,14	PPFIA4	-1,22
TNFAIP3	-0,15	SLC2A3	-1,22
PFKL	-0,15	HK2	-1,24
SLC2A1	-0,16	PLAUR	-1,25
LOX	-0,16	SCARB1	-1,25
FOS	-0,19	SIAH2	-1,30
EXT1	-0,19	ANGPTL4	-1,30
RBPJ	-0,22	PYGM	-1,32
PRKCA	-0,22	TGFBI	-1,34
NEDD4L	-0,23	PDGFB	-1,34
STBD1	-0,24	GLRX	-1,40
LDHA	-0,24	TMEM45A	-1,49
HS3ST1	-0,26	VEGFA	-1,50
SDC4	-0,26	DDIT3	-1,52
ETS1	-0,27	BGN	-1,59
PFKFB3	-0,27	MXI1	-1,62
ALDOA	-0,28	PPP1R3C	-1,68
CDKN1A	-0,30	S100A4	-1,71
BHLHE40	-0,30	RRAGD	-1,79
SDC3	-0,32	PGF	-1,95
PDK1	-0,35	IER3	-1,99
ENO3	-0,36	NDRG1	-2,00
DPYSL4	-0,37	IGFBP3	-2,07
PFKP	-0,37	HOXB9	-2,26
ANKZF1	-0,39	DDIT4	-2,43
MAFF	-0,39	GCK	-2,50
PPP1R15A	-0,40	PCK1	-2,83
GAPDH	-0,40	PLIN2	-3,64
HK1	-0,42	PLAC8	-5,04
GYS1	-0,42	UGP2	0,01
GPI	-0,42	IDS	0,03
CSRP2	-0,43	PGM2	0,04
SULT2B1	-0,43	ADORA2B	0,06
TPI1	-0,43	GPC1	0,07
KLHL24	-0,43	NR3C1	0,08
PRDX5	-0,44	ZFP36	0,08
TPBG	-0,45	HEXA	0,09
CCNG2	-0,45	AKAP12	0,09
SELENBP1	-0,46	NAGK	0,09
GAA	-0,46	CDKN1B	0,09
PHKG1	-0,49	MT2A	0,11
HSPA5	-0,50	KIF5A	0,12
EFNA1	-0,50	CHST3	0,13
KDELR3	-0,52	CDKN1C	0,14
TKTL1	-0,52	HMOX1	0,14
XPNPEP1	-0,54	GPC4	0,15
SDC2	-0,55	CITED2	0,16
PAM	-0,55	ANXA2	0,17
JMJD6	-0,56	VHL	0,18
SLC37A4	-0,56	ATP7A	0,21
TPD52	-0,58	PPARGC1A	0,21
PNRC1	-0,61	DTNA	0,25
BNIP3L	-0,61	KLF7	0,25
TES	-0,63	ZNF292	0,27
ERRFI1	-0,63	NDST1	0,28
BCAN	-0,64	CASP6	0,28
CP	-0,66	F3	0,40
TIPARP	-0,69	VLDLR	0,40
ENO1	-0,70	GCNT2	0,41
P4HA2	-0,71	MYH9	0,45
NDST2	-0,71	HDLBP	0,46
INHA	-0,73	RORA	0,46
FOSL2	-0,73	BCL2	0,49
IL6	-0,75	MIF	0,50
P4HA1	-0,75	EGFR	0,55
AMPD3	-0,79	TGFB3	0,59
PGK 1,00	-0,80	FOXO3	0,64
BTG1	-0,84	CA12	0,78
STC1	-0,90	CXCR4	1,07
		SERPINE1	1,52
		HAS1	1,96
		MT1E	3,30

B.

Hallmark Hypoxia

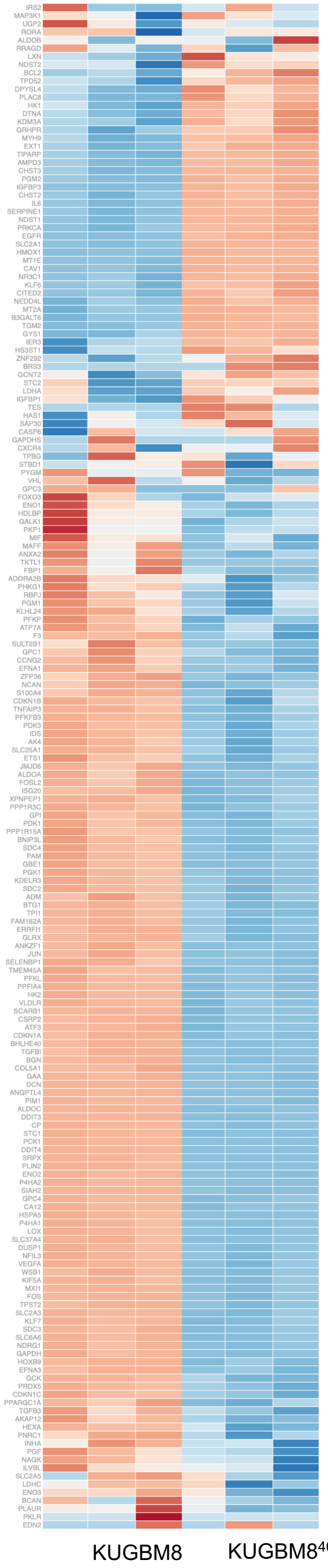

Gene	Log$_2$(FC)	Gene	Log$_2$(FC)
GPC1	0,00	VLDLR	-1,76
HDLBP	0,00	SCARB1	-1,84
FOXO3	-0,01	BHLHE40	-1,88
PLAUR	-0,01	CA12	-1,92
ANXA2	-0,03	SLC2A3	-1,95
VHL	-0,06	SRPX	-1,96
ILVBL	-0,07	ISG20	-2,01
PGF	-0,09	FAM162A	-2,03
NAGK	-0,10	PPP1R3C	-2,09
ENO1	-0,11	PIM1	-2,14
PGM1	-0,15	PLIN2	-2,22
GPI	-0,17	MXI1	-2,22
HEXA	-0,18	PKP1	-2,41
KLHL24	-0,18	PPFIA4	-2,42
SLC2A5	-0,20	GAA	-2,49
PNRC1	-0,23	P4HA1	-2,52
ADORA2B	-0,23	CDKN1C	-2,54
GALK1	-0,26	HSPA5	-2,58
ATP7A	-0,26	KIF5A	-2,60
RBPJ	-0,27	NCAN	-2,62
TGFB3	-0,27	CP	-2,79
PFKP	-0,30	EFNA3	-2,93
ENO3	-0,32	GCK	-2,96
ADM	-0,33	ENO2	-3,16
F3	-0,34	CSRP2	-3,26
XPNPEP1	-0,36	GPC4	-3,45
PPP1R15A	-0,38	BGN	-3,48
TNFAIP3	-0,39	DDIT3	-3,62
FOSL2	-0,40	FBP1	-4,08
TGFBI	-0,40	DDIT4	-4,08
MAFF	-0,43	ALDOC	-4,13
JUN	-0,46	TKTL1	-4,21
CDKN1B	-0,47	DCN	-4,42
SLC25A1	-0,49	SULT2B1	-4,67
PFKFB3	-0,49	HOXB9	-5,23
SDC3	-0,49	SELENBP1	-5,68
PYGM	-0,50	ANGPTL4	-5,73
ETS1	-0,50	UGP2	0,08
AKAP12	-0,51	TPBG	0,08
CCNG2	-0,52	RORA	0,11
TPI1	-0,53	RRAGD	0,11
PRDX5	-0,54	IRS2	0,11
GAPDH	-0,57	STBD1	0,15
BNIP3L	-0,65	STC2	0,18
IDS	-0,65	CASP6	0,18
TPST2	-0,66	LDHA	0,21
NDRG1	-0,70	MAP3K1	0,24
JMJD6	-0,72	SAP30	0,31
SLC6A6	-0,77	DPYSL4	0,31
COL5A1	-0,78	ZNF292	0,33
PDK3	-0,81	HK1	0,34
S100A4	-0,86	GRHPR	0,39
DUSP1	-0,89	DTNA	0,40
AK4	-0,90	CITED2	0,41
PDK1	-0,90	EXT1	0,43
PPARGC1A	-0,96	KDM3A	0,43
PHKG1	-0,97	MYH9	0,44
PAM	-0,98	KLF6	0,55
PFKL	-0,99	GCNT2	0,57
VEGFA	-1,02	HAS1	0,61
GBE1	-1,06	GYS1	0,63
ANKZF1	-1,07	NEDD4L	0,68
ZFP36	-1,08	HS3ST1	0,71
LDHC	-1,09	PGM2	0,76
HK2	-1,10	B3GALT6	0,78
ERRFI1	-1,11	BCL2	0,81
INHA	-1,11	CXCR4	0,92
GLRX	-1,14	IGFBP3	1,02
SLC37A4	-1,15	NDST2	1,11
KLF7	-1,19	NR3C1	1,11
ALDOA	-1,20	PRKCA	1,11
KDELR3	-1,21	IL6	1,12
SDC4	-1,23	TIPARP	1,14
EFNA1	-1,24	CHST3	1,17
CDKN1A	-1,26	TPD52	1,20
TMEM45A	-1,27	SLC2A1	1,31
BCAN	-1,29	AMPD3	1,33
FOS	-1,31	IER3	1,37
SIAH2	-1,34	LXN	1,54
SDC2	-1,42	HMOX1	1,56
NFIL3	-1,42	IGFBP1	1,57
ATF3	-1,46	NDST1	1,59
BTG1	-1,50	CHST2	1,70
MIF	-1,53	EGFR	1,85
LOX	-1,54	CAV1	2,07
PGK 1,00	-1,57	MT2A	2,82
P4HA2	-1,64	SERPINE1	2,95
STC1	-1,69	TGM2	3,28
WSB1	-1,72	PLAC8	3,98
		MT1E	4,37

Supplementary Figure S3. **A-B**. Differential expression heat map of Hallmark Hypoxia gene set for U373 **(A)** and KUGBM8 **(B)** cells and DEG scores for each gene.

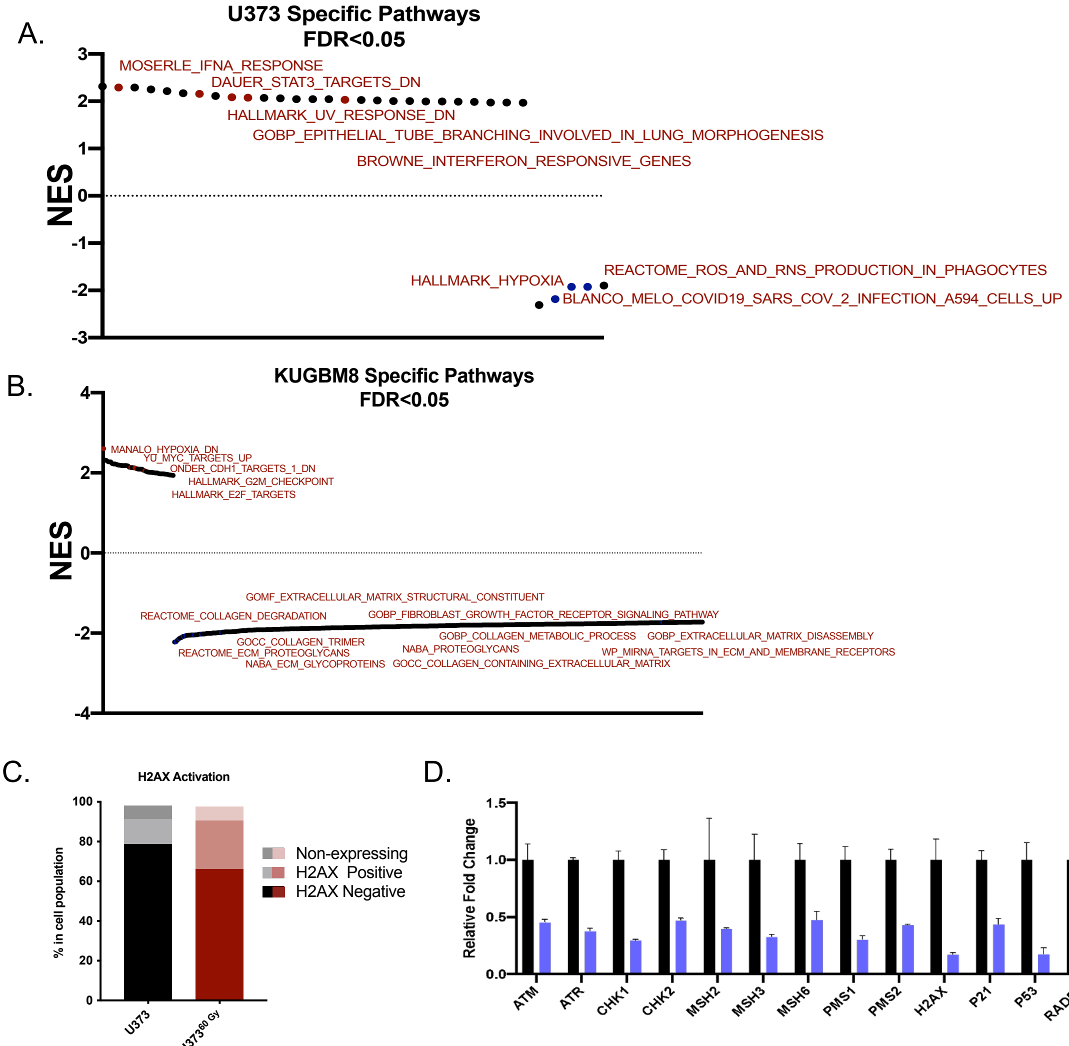

Supplementary Figure S4. **A.** Representation of U373-specific differentially enriched pathways (FDR cut-off <0.05) **B.** Representation of KUGBM8-specific differentially enriched pathways (FDR cut-off <0.05) **C.** Basal H2AX activation levels of U373 and U373$^{60\ Gy}$ cells. **D.** qRT-PCR results showing gene expression levels of different DNA damage response and repair elements in KUGBM8 cells.

GOBP Regulation of DNA Repair

Gene	Log_2(FC)	Gene	Log_2(FC)
PARG	0,00	SHLD1	0,10
EYA4	0,00	RAD51	0,10
BABAM2	-0,01	TWIST1	0,11
PARP3	-0,02	UBR5	0,11
RNF168	-0,02	NSD2	0,12
UIMC1	-0,02	H2AX	0,12
MORF4L1	-0,02	UBE2V1	0,13
SIRT1	-0,03	FMN2	0,13
VPS72	-0,03	POT1	0,13
PARPBP	-0,03	WDR48	0,14
HDGFL2	-0,03	OTUB1	0,14
UBE2N	-0,04	KDM4D	0,15
KDM1A	-0,05	TFIP11	0,16
MRNIP	-0,05	HELB	0,16
CDK9	-0,05	OGG1	0,16
SPIRE1	-0,06	RPA2	0,18
ERCC8	-0,06	TERF2IP	0,18
WAS	-0,07	ARID2	0,19
SIRT6	-0,07	RNF169	0,20
RAD51AP1	-0,09	CUL4A	0,20
YEATS4	-0,10	ACTR2	0,21
RNF8	-0,10	ERCC6	0,21
MORF4L2	-0,14	DEK	0,23
UBQLN4	-0,15	CHEK1	0,23
TIGAR	-0,15	BRD8	0,23
RUVBL2	-0,16	PPP4R2	0,23
MMS19	-0,18	SPIRE2	0,23
AXIN2	-0,20	FOXM1	0,24
MAGEF1	-0,22	BRCA1	0,24
SHLD3	-0,22	SHLD2	0,25
PNKP	-0,23	RTEL1	0,26
HSF1	-0,25	POLH	0,26
XRCC1	-0,26	EPC1	0,27
MAD2L2	-0,29	POGZ	0,28
ACTL6A	-0,30	TRIM28	0,28
SPIDR	-0,33	EYA3	0,29
UBE2V2	-0,34	SMCHD1	0,29
ZNF365	-0,37	NUDT16L1	0,31
MIR221	-0,40	BRCC3	0,32
SLF1	-0,41	RAD52	0,32
MRGBP	-0,47	FANCB	0,32
WRNIP1	-0,48	DHX9	0,33
AUNIP	-0,50	TP53BP1	0,36
RMI2	-0,50	PPP4R3B	0,37
WRAP53	-0,50	FAM168A	0,39
RPS3	-0,51	POLQ	0,40
TMEM161A	-0,53	ING3	0,43
KMT5C	-0,56	SETD2	0,43
OTUB2	-0,64	TRRAP	0,44
CEBPG	-0,78	EP400	0,45
KLHL15	-0,79	ACTB	0,48
PRKCG	-1,24	PML	0,51
MEAF6	0,00	HDAC10	0,53
PRKDC	0,01	EGFR	0,55
CYREN	0,01	SLF2	0,55
KMT5B	0,01	DTX3L	0,57
PPP4C	0,01	PARP9	0,59
DDX11	0,01	SETMAR	0,65
FBH1	0,02	NPAS2	0,66
USP1	0,03	PCNA	0,74
MBTD1	0,03	ZCWPW1	0,75
TIMELESS	0,04	TEX15	0,76
PARP1	0,04	RIF1	0,78
RECQL5	0,05	TRIP12	0,85
BABAM1	0,05	FUS	0,88
RUVBL1	0,06	EPC2	0,89
KAT5	0,06	HMGA2	1,08
TERF2	0,08	EYA2	1,24
SIRT7	0,08	EYA1	1,37
CCDC117	0,08	RADX	2,59
HMGB1	0,09	MGMT	5,26
DMAP1	0,09		
RBBP8	0,09		
KAT7	0,09		
FIGNL1	0,10		

Reactome DNA DSB Repair

Gene	Log_2(FC)	Gene	Log_2(FC)
EYA4	0,00	DNA2	0,11
BABAM2	-0,01	RFC1	0,12
RAD51C	-0,02	NSD2	0,12
RNF168	-0,02	UBC	0,12
UIMC1	-0,02	H2AX	0,12
POLD2	-0,03	MUS81	0,13
UBE2N	-0,04	H2BC9	0,13
H4C14	-0,04	BRIP1	0,14
UBE2I	-0,05	XRCC6	0,14
NBN	-0,06	H4-16	0,14
H4C8	-0,07	FEN1	0,15
TDP1	-0,07	EXO1	0,17
SIRT6	-0,07	HUS1	0,17
POLD3	-0,08	RPA2	0,18
H4C5	-0,09	PIAS4	0,18
RAD51AP1	-0,09	NHEJ1	0,18
RNF8	-0,10	BAP1	0,19
TOP3A	-0,12	RAD1	0,19
XRCC3	-0,15	EME2	0,19
POLD1	-0,15	BLM	0,20
UBXN1	-0,15	H2BC21	0,21
PARP2	-0,16	MAPK8	0,22
H2BC5	-0,17	POLE	0,22
ERCC1	-0,17	CHEK1	0,23
ABRAXAS1	-0,18	RPA3	0,23
POLK	-0,19	RFC3	0,23
LIG3	-0,19	BAZ1B	0,23
UBA52	-0,19	PPP4R2	0,23
RPS27A	-0,20	BRCA1	0,24
RAD9A	-0,22	H2BC17	0,25
H2BC15	-0,24	TDP2	0,25
DCLRE1C	-0,24	RMI1	0,25
RAD17	-0,25	RNF4	0,26
POLM	-0,25	RTEL1	0,26
RPA1	-0,25	H2BC4	0,26
XRCC1	-0,26	POLH	0,26
UBB	-0,26	SUMO2	0,27
KDM4A	-0,27	RFC5	0,28
H2BC8	-0,27	EYA3	0,29
ABL1	-0,29	H2BC11	0,30
KDM4B	-0,32	BRCA2	0,31
SPIDR	-0,33	BRCC3	0,32
RAD51B	-0,33	TOPBP1	0,32
UBE2V2	-0,34	RAD52	0,32
RFC4	-0,42	PALB2	0,33
CHEK2	-0,45	LIG4	0,35
EME1	-0,46	TP53BP1	0,36
POLL	-0,48	SUMO1	0,37
RMI2	-0,50	KPNA2	0,39
XRCC4	-0,51	CCNA2	0,40
POLD4	-0,58	POLQ	0,40
H2BU1	-0,63	SMARCA5	0,40
APBB1	-0,82	TIPIN	0,40
H2BC7	-1,36	ATM	0,41
ERCC4	0,00	GEN1	0,42
MRE11	0,00	WRN	0,44
PRKDC	0,01	RAD50	0,46
RAD51D	0,01	HERC2	0,48
PPP4C	0,01	CLSPN	0,55
POLE2	0,01	H4C9	0,59
CDK2	0,03	XRCC5	0,60
XRCC2	0,04	H2BC6	0,64
RFC2	0,04	ATR	0,66
TIMELESS	0,04	PCNA	0,74
PARP1	0,04	BARD1	0,76
BABAM1	0,05	RIF1	0,78
KAT5	0,06	H2BC10	0,81
MDC 1,00	0,07	H2BC12	1,01
POLE3	0,07	H4C3	1,07
TP53	0,07	SLX1B	1,18
ATRIP	0,08	EYA2	1,24
PAXIP1	0,08	EYA1	1,37
PPP5C	0,08	H2BC13	1,37
RHNO1	0,09	RAD9B	1,49
RBBP8	0,09	CCNA1	1,77
SLX4	0,10	H4C4	3,18
RAD51	0,10		
POLE4	0,11		

GOBP Regulation of DNA Repair

Gene	Log_2(FC)	Gene	Log_2(FC)
UBQLN4	0,00	RNF169	0,21
BABAM1	-0,01	RPS3	0,23
CCDC117	-0,01	PARG	0,23
UBE2V2	-0,01	SPIRE1	0,24
PARP1	-0,02	PARP3	0,24
ACTR2	-0,05	DDX11	0,25
KDM4D	-0,05	OTUB1	0,26
PARP9	-0,06	SLF1	0,26
TIGAR	-0,07	RNF8	0,26
TERF2	-0,07	DEK	0,28
PNKP	-0,12	UBE2N	0,29
RUVBL2	-0,13	SPIRE2	0,30
POT1	-0,13	ERCC8	0,31
SHLD3	-0,14	SETD2	0,32
WRNIP1	-0,17	RAD51AP1	0,33
FBH1	-0,17	XRCC1	0,35
NUDT16L1	-0,19	ACTL6A	0,36
MORF4L1	-0,20	OGG1	0,37
SPIDR	-0,24	PARPBP	0,37
SIRT6	-0,25	SETMAR	0,39
UIMC1	-0,28	DMAP1	0,42
EYA3	-0,30	NSD2	0,42
POGZ	-0,30	KMT5B	0,44
KDM1A	-0,30	RBBP8	0,45
MRGBP	-0,31	HMGB1	0,49
RUVBL1	-0,34	RAD51	0,51
TERF2IP	-0,35	YEATS4	0,51
PPP4R2	-0,39	TFIP11	0,52
VPS72	-0,40	ACTB	0,54
FAM168A	-0,41	SHLD2	0,55
MMS19	-0,43	USP1	0,57
HSF1	-0,45	CUL4A	0,67
HDAC10	-0,46	PML	0,67
HDGFL2	-0,46	CGAS	0,68
HELB	-0,46	BRD8	0,69
PPP4C	-0,49	ARID2	0,70
CDK9	-0,57	DTX3L	0,71
RAD52	-0,59	AUNIP	0,72
MAD2L2	-0,60	RIF1	0,79
MORF4L2	-0,63	EPC1	0,80
TWIST1	-0,63	ZCWPW1	0,83
ZNF365	-0,64	PRKDC	0,84
KLHL15	-0,76	TRIM28	0,85
SHLD1	-0,77	PPP4R3B	0,87
UBE2V1	-0,79	TRIP12	0,87
KMT5C	-0,79	EYA4	0,87
RMI2	-0,80	PCNA	0,88
TMEM161A	-0,88	CHEK1	0,90
BABAM2	-0,92	TIMELESS	0,99
RNF168	-0,93	RTEL1	1,00
CEBPG	-1,02	FOXM1	1,00
MAGEF1	-1,13	BRCA1	1,02
AXIN2	-1,13	TRRAP	1,03
EYA1	-1,14	SLF2	1,11
NPAS2	-1,74	FMN2	1,12
MGMT	-1,78	BRCC3	1,13
OTUB2	-1,84	EP400	1,17
WAS	-2,01	SMCHD1	1,22
PRKCG	-3,15	TEX15	1,23
FGF10	-5,59	SIRT1	1,24
WRAP53	0,02	ING3	1,31
MBTD1	0,05	FUS	1,37
RECQL5	0,05	ERCC6	1,40
UBR5	0,06	H2AX	1,41
POLQ	0,08	KAT5	1,41
SIRT7	0,09	CYREN	1,53
TP53BP1	0,11	USP51	1,76
POLH	0,12	FIGNL1	1,78
RPA2	0,13	EPC2	1,82
MEAF6	0,14	DHX9	1,83
KAT7	0,14	EGFR	1,85
MRNIP	0,16	MIR221	2,69
WDR48	0,18	HMGA2	3,04
FANCB	0,20	RADX	6,67

Reactome DNA DSB Repair

Gene	Log_2(FC)	Gene	Log_2(FC)
BABAM1	-0,01	PIAS4	0,27
UBE2V2	-0,01	MRE11	0,28
MAPK8	-0,02	KDM4A	0,29
PARP1	-0,02	H2BC5	0,29
UBB	-0,05	RHNO1	0,29
POLM	-0,05	UBE2N	0,29
POLK	-0,07	RAD9A	0,29
BAP1	-0,08	KPNA2	0,32
SUMO1	-0,09	RAD17	0,32
RAD9B	-0,09	H4C4	0,33
UBC	-0,13	H2BC17	0,33
UBE2I	-0,14	RAD51AP1	0,33
POLL	-0,15	XRCC1	0,35
POLD1	-0,15	RFC3	0,37
POLD3	-0,16	PALB2	0,39
SUMO2	-0,19	RMI1	0,39
ERCC1	-0,20	NSD2	0,42
POLD4	-0,22	UBXN1	0,42
SPIDR	-0,24	XRCC6	0,43
SIRT6	-0,25	TDP1	0,44
RPA1	-0,28	POLD2	0,44
H4C5	-0,28	XRCC5	0,45
UIMC1	-0,28	RBBP8	0,45
RAD1	-0,29	PPP5C	0,47
RFC4	-0,29	TIPIN	0,50
EYA3	-0,30	RPS27A	0,50
RAD51B	-0,35	RAD51	0,51
PPP4R2	-0,39	BARD1	0,57
H2BC15	-0,44	H4C14	0,59
H2BC9	-0,46	TOPBP1	0,60
TOP3A	-0,47	POLE2	0,61
PPP4C	-0,49	WRN	0,73
KDM4B	-0,57	FEN1	0,73
RFC1	-0,57	POLE	0,74
RAD52	-0,59	RIF1	0,79
EME1	-0,59	BAZ1B	0,80
H2BC7	-0,60	ABRAXAS1	0,82
XRCC4	-0,60	MUS81	0,83
PARP2	-0,63	HUS1	0,83
CCNA1	-0,73	ATM	0,84
CDK2	-0,78	PRKDC	0,84
RMI2	-0,80	EYA4	0,87
XRCC3	-0,81	RAD51D	0,87
BABAM2	-0,92	CCNA2	0,87
RNF168	-0,93	PCNA	0,88
APBB1	-1,02	DCLRE1C	0,89
CHEK2	-1,04	BLM	0,89
H2BC10	-1,09	CHEK1	0,90
H2BC21	-1,13	POLE4	0,92
EYA1	-1,14	ATRIP	0,95
LIG3	-1,20	HERC2	0,97
H2BC8	-1,49	TP53	0,97
H2BC13	-1,55	EME2	0,99
UBA52	0,01	TIMELESS	0,99
ERCC4	0,01	RTEL1	1,00
SMARCA5	0,04	BRCA1	1,02
ABL1	0,06	BRIP1	1,07
POLQ	0,08	CLSPN	1,09
RFC2	0,10	H4-16	1,11
TP53BP1	0,11	ATR	1,12
SLX4	0,12	PAXIP1	1,13
POLH	0,12	BRCC3	1,13
RPA2	0,13	NBN	1,22
RPA3	0,14	DNA2	1,33
RAD51C	0,14	BRCA2	1,37
RFC5	0,15	H2AX	1,41
LIG4	0,17	KAT5	1,41
H4C3	0,18	RAD50	1,43
TDP2	0,20	H4C9	1,48
POLE3	0,20	XRCC2	1,57
EXO1	0,22	H2BC11	1,82
RNF4	0,23	H2BC12	2,02
MDC 1,00	0,26	NHEJ1	2,03
RNF8	0,26	GEN1	2,27
		H4C8	2,34
		H2BC4	3,01

Supplementary Table S1. A-B. DEG scores list of GOBP Regulation of DNA Repair **(A)** and Reactome DNA DSB Repair **(B)** gene sets for U373 cells. **C-D**. DEG Scores list of GOBP Regulation of DNA Repair **(C)** and Reactome DNA DSB Repair **(D)** gene sets for KUGBM8 cells.

Gene	Sequence of Forward Primer (5’-3’)	Sequence of Reverse Primer (5’-3’)
GAPDH	AGCCACATCGCTCAGACAC	GCCCAATACGACCAAATCC
ATM	CATCGCATGTGATTAAAGCA	TTCTGATAGGAATCAGGGC
ATR	CAATTGTGGAGGAGATTTCC	CTTCTGAGAACTCTTGTATCTG
CHK1	CGGTATAATAATCGTGAGCG	TTCCAAGGGTTGAGGTATGT
CHK2	CTCGGGAGTCGGATGTTGAG	GAGTTTGGCATCGTGCTGGT
MSH2	TTGATGGCCAGAGACAGGTT	CCATGTCTCCAGCAGTCTCT
MSH3	CCATCATGGCTCAGATTGGC	ATCATGAGTGCTCGTCCCTC
MSH6	ACTTCAGCAGGGACGTAACA	ACTTCAGCAGGGACGTAACA
PMS1	ATGAGTGGAGCAGGGGAAAT	CACTTGCTGACATGGGTTTCT
PMS2	ACTTCCGTGGATTCTGAGGG	GTGTTTGGGGTTGCGAGATT
MLH1	CTACTTCCAGCAACCCCAGA	AGAACCTCATGTCCCTGCTC
H2AX	ACTCAACTGGGCAATCCAAG	GGGTTAGCTGCAGAATTCCA
p21	GGCAGACCAGCATGACAGATTT	AAGATGTAGAGCGGGCCTTTGA
TP53	GGTGACACGCTTCCCTGGATT	AGGGGGACAGAACGTTGTTTTCAG
RAD51	TGCGGACCGAGTAATGGCA	TCCTTCTTTGGCGCATAGGCA
VEGFA	TGTGAATGCAGACCAAAGAAAGA	ACCAACGTACACGCTCCAG
MGMT	GGATTGCCTCTCATTGCTCC	CCCGTTTTCCAGCAAGAGTC

Supplementary Table S2. List of primers and sequences used in qRT-PCR

Antibody	Brand/Catalog Number
**Phospho-ATR Ser428**	Cell Signaling/5161
**ATR Total**	Cell Signaling/2790
**Phospho-ATM Ser1981**	Cell Signaling/13050
**ATM total**	Cell Signalling/11G12
**Phospho-CHK1 Ser345**	Cell Signalling/133D3
**CHK1**	Cell Signalling/23605
**Phospho-CHK2 Ser19**	Cell Signalling/2666
**CHK2**	Cell Signalling/2662S
**γH2AX**	EMD Millipore/05-636
**RAD51**	Abcam/ab213
**MSH2**	Abcam/ab52266
**MSH6**	Abcam/ab92471
**PMS1**	Abcam/ab129020
**PMS2**	Abcam/ab110638
**MLH1**	Abcam/ab92312
**MSH3**	Abcam/ab111107
**α-tubulin**	Abcam/ab15246
**GAPDH**	Abcam/ab9485

Supplementary Table S3. List of primary antibodies used in Western Blot

Antibody	Brand/ Catalog Number
**Anti-vinculin**	Abcam/ab73412
**Rhodamin-phalloidin**	Thermo R415
**Mounting medium with DAPI**	Abcam/ab104139
**Phosphor-Histone H2A.X (Ser139)**	Millipore/05-636
**53BP1**	Novus/NB100-304
**Alexa Fluor® 488 Anti- IgG Secondary Antibody**	Invitrogen
**Alexa Fluor® 594 Anti- IgG Secondary Antibody**	Invitrogen

Supplementary Table S4. List of primary and secondary antibodies used in immunofluorescence staining
